# Decoding the Longevity Networks of the Mediterranean Diet: Systems Biology and Multi-Pathway Mechanisms Shaping Healthspan

**DOI:** 10.3390/ijms27083634

**Published:** 2026-04-19

**Authors:** Sandra K. Szlapinski, Bryana Hallam, Andrew Charrette, Najla Guthrie, Corey J. Hilmas

**Affiliations:** Division of Client Services, Department of Regulatory Affairs, KGK Science Inc., London, ON N6B 3L1, Canada

**Keywords:** mediterranean diet, longevity, polyphenols, DailyColors^TM^, cognition, metabolic health

## Abstract

The Mediterranean Diet (MD) is recognized for promoting longevity and reducing the risk of chronic disease, yet the mechanisms underlying these benefits remain uncharacterized. This review highlights the diverse nutritional and phytoactive constituents of the MD and research exploring its complex network of polyphenols. It discusses data evaluating MD-derived constituents formulated into a dietary supplement capsule developed using a systems and network biology framework. Component selection was based on their actions on enzyme systems involved in senescence-related pathways and health preservation. This review highlights how MD components synergistically modulate pathways central to antioxidant activity, cognitive health, and aging. Liquid chromatography–mass spectrometry identified phytochemically diverse constituents in capsules (supplied by DailyColors™, Warwickshire, UK and Sebastopol, CA, USA) derived from primary color groups in sixteen Mediterranean plants. These constituents were mapped to bioactive networks targeting enzymes linked to inflammation, metabolic regulation, and cellular senescence. Preclinical studies demonstrated the modulation of mitochondrial and metabolic health markers, with complementary effects on cytokine inhibition and glucose sensitivity. Two clinical studies confirmed broad proteomic and epigenetic effects on pathways governing immunity, skeletal muscle, cognition, and inflammation. Therefore, this review advances a novel perspective that MD polyphenols act through synergistic, multi-pathway interactions that link dietary patterns to coordinated regulation of longevity and healthy aging.

## 1. Introduction

The Mediterranean Diet (MD), long practiced in cultures around the Mediterranean Sea, is one of the most well-recognized and copied health-promoting dietary patterns. The diet emphasizes a “rainbow” of colorful fruits and vegetables, whole grains, legumes, olive oil as the primary fat, in concert with a moderate intake of fish, dairy, and red wine, and low red meat intake. It is widely promoted for weight management, reduction in risk factors for developing common chronic diseases, and longevity support [1,2,3]. Its benefits stem from both nutritive components and abundant phytochemicals that contribute to its anti-inflammatory and antioxidant properties, including polyphenols [2,4,5].

The MD is among the most consistently validated models for extending health span, yet its mechanisms remain difficult to fully define due to the complexity and diversity of its bioactive compounds. Therefore, this review will discuss some of the available evidence for select polyphenols in the MD. This review will also discuss the approach, rationale and scientific value for using a dietary supplement under controlled conditions in a clinical trial to best approximate ingestion of the MD in a generally healthy population. This strategy was implemented to overcome costs, and logistical and compliance barriers with strict adherence to an MD lifestyle that would be normalized across the entire study population. While cost and access can be significant barriers to maintaining the MD lifestyle year-round, a complex formulation incorporating select, powerful constituents of the MD in dietary supplement capsule form allows for greater compliance and reduced variability during investigation of these polyphenols in preclinical and clinical protocol designs. A supplement format allows for controlled unit dosing to study wide-ranging benefits and decode the mechanisms by which the MD is associated with beneficial health effects. By studying the combined entourage effect of beneficial components from diverse-colored Mediterranean fruits, vegetables, herbs, and spices through preclinical (e.g., biochemical and cellular mechanisms) and clinical research (e.g., efficacy in humans), these studies have provided a mechanistic map of how diet-derived polyphenol diversity influences antioxidant potential for longevity, maintenance of cognitive health, and the overall aging process.

## 2. The Potential of the Mediterranean Diet to Reverse Aging and Disease

Cellular aging involves the gradual loss of structural and functional integrity, with telomere length as a key biomarker [1]. Multiple mechanisms contribute, including genomic instability, telomere and mitochondrial dysfunction, impaired autophagy, inflammation, and oxidative stress [6,7], and current research aims to identify interventions that can slow or reverse these processes. Poor nutrition also accelerates telomere shortening and reduces telomerase activity [8], while diets rich in fruits, vegetables, fish, and especially the MD, are associated with longer telomeres, higher telomerase activity, lower inflammatory cytokines, and reduced oxidative stress [9]. Because the MD provides strong anti-inflammatory and antioxidant effects, it is considered a promising non-pharmacological strategy to mitigate age-related decline.

In fact, clinical evidence links MD adherence to reduced all-cause and cardiovascular mortality as well as slower cognitive decline [10,11]. Polyphenols, key MD phytonutrients, likely drive many of these benefits through anti-inflammatory, antioxidant, and epigenetic mechanisms [12]. Human and animal studies show that higher polyphenol intake reduces inflammation, boosts antioxidant defenses, improves cognition, and influences cell signaling and gene expression [13,14,15,16,17,18]. The MD may also modulate extracellular vesicles (EVs), which are cellular stress surrogates linked to aging [19]. MD interventions have reduced EV counts and prothrombotic vesicle release in older adults and high-risk individuals [20,21].

**Figure 1:**
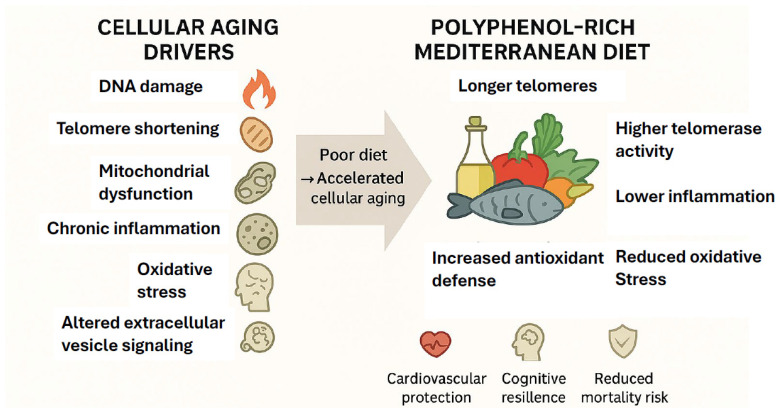
Mediterranean Diet modulation of cellular aging mechanisms.

At the molecular systems level, many of the anti-aging effects attributed to MD-derived polyphenols converge on the maintenance of cellular nicotinamide adenine dinucleotide (NAD^+^) pools, a central metabolic cofactor that coordinates energy metabolism, stress adaptation, and genome stability [22,23,24]. NAD^+^ is an obligate substrate for sirtuins (SIRT), a family of NAD^+^-dependent deacetylases that regulate mitochondrial biogenesis, inflammation, telomere integrity, and DNA damage responses [25,26]. Increased NAD^+^ availability enhances SIRT activity, particularly SIRT1 and SIRT6, thereby promoting DNA repair through activation of base excision repair pathways, stabilization of telomeric chromatin, and suppression of pro-aging inflammatory signaling [27,28]. In parallel, NAD^+^ supports the activity of poly (ADP-ribose) polymerases (PARPs), which function as DNA damage sensors essential for initiating repair following strand breaks [29]. Age-associated NAD^+^ decline disrupts this integrated network, resulting in impaired DNA repair capacity, mitochondrial dysfunction, and accelerated cellular senescence [30]. Collectively, these observations suggest that dietary patterns and nutraceutical strategies capable of preserving or restoring NAD^+^ homeostasis may exert pleiotropic, system-wide benefits by synchronizing metabolic resilience, genomic integrity, and epigenetic regulation, providing a unifying mechanistic framework for MD-associated longevity effects. A summary of the effects of the MD on the various cellular aging mechanisms is presented in Figure 1.

Despite strong evidence for MD-mediated health and longevity benefits, its mechanistic complexity, in relation to its synergistic interactions among the diverse plant compounds, and practical barriers (e.g., cost, availability, cultural factors, and adherence) limit widespread adoption [31]. Therefore, alternative methods to optimally administer the numerous benefits contained in the MD while increasing access to those beneficial phytonutrients are important. One approach is using easy-to-consume, unit-dosed nutraceuticals designed to provide diverse MD-derived polyphenols in a simple, accessible formulation. The choice for polyphenol inclusion into a dietary supplement was daunting because there are so many diverse food choices in the MD; however, the ones selected for inclusion in DailyColors^TM^ were based upon a very careful and methodological approach.

## 3. Critical Components of the MD Formulated into a Capsule

The MD has been widely regarded as one of the healthiest dietary patterns [32], yet efforts to isolate a single “active” polyphenol have failed to replicate its broad benefits. Most individual compounds show promise in preclinical models but cannot reproduce the MD’s systemic effects in humans. Nonetheless, advances in analytical chemistry method detection and bioinformatics, and application of integrative and network biology approaches to the MD have enabled formulation of dietary supplements with unique polyphenol profiles. The dietary supplement selected for this review is a proprietary blend of specific polyphenols imparting individualized color and functional properties to their fruit and vegetable sources. These polyphenols are thought to operate together to downregulate enzyme activity in critical pathways involved in the regulation of healthy aging.

The process of careful polyphenol selection began with a liquid chromatography–mass spectrometry (LC-MS) mapping of their diversity from red, green, blue, yellow and orange produce found in the MD. The DailyColors™ polyphenol-rich blend is a blend of powders and extracts from 16 unique produce typically consumed as part of the MD. The dietary supplement was formulated from apple, pomegranate, tomato, beet, olive, rosemary, green coffee bean (e.g., green coffee bean extract, GCBE), kale, onion, ginger, grapefruit, carrot, grape, blueberry, currant, and elderberry, encompassing well over 150 highly bio-active compounds isolated from the polychromatic representations of these botanicals (Table 1).

The food components selected in this MD-inspired dietary supplement impart a more sustainable, multifaceted blend and multidirectional pathway model to mirror the ideal nutrition standard of the MD. Using LC-MS, the company mapped hundreds of distinct phytochemicals, including polyphenols, across 16 Mediterranean botanicals, identifying unique ‘color signatures’ that correlate with biological activity. These molecules collectively target overlapping networks involved in numerous physiological functions such as oxidative stress, immune modulation, mitochondrial biochemistry, and cell-senescence regulation. The 16 diverse foods were carefully selected based on published mechanisms of action for benefits to health and aging, which will be described in more detail in Section 4.

The resulting formulation was further refined after screening a multitude of Mediterranean phytochemical complexes against multiple enzyme systems implicated in chronic disease and aging. The literature identified strong modulatory activity toward Cluster of Differentiation (CD) 38, CD73, Cathepsin S, Beta-site Amyloid Precursor Protein Cleaving Enzyme 1 (BACE1), Cyclin-dependent kinase 5 (CDK5), and Janus kinase (Jak) 1–3, which are key regulatory enzymes in metabolic resilience, neuro-inflammation, and immune signaling. CD38 was of particular interest, as its age-related increase drives NAD^+^ decline [33], and the blend showed potent CD38 inhibition in preclinical assays (see Section 3.1). Other preclinical work explored the cellular pathways influenced by the blend, while numerous clinical studies already document the individual health effects of many of its components. The efficacy of the polyphenol blend in this MD-inspired dietary supplement has also been investigated in two separate clinical trials.

Thus, while explicit numeric cutoffs (e.g., percent inhibition thresholds) were not applied at the initial formulation stage, selection was quantitatively constrained through analytical validation (HPLC), comparative enzyme inhibition assays, and a reproducible demonstration of synergistic activity across multiple biological targets, providing a rigorous and defensible methodological framework consistent with systems-level nutritional biology.

### 3.1. Cellular and Organismal Mechanisms of an MD Supplement: Preclinical Data

#### 3.1.1. Enzyme Inhibition Assays

**Figure 2:**
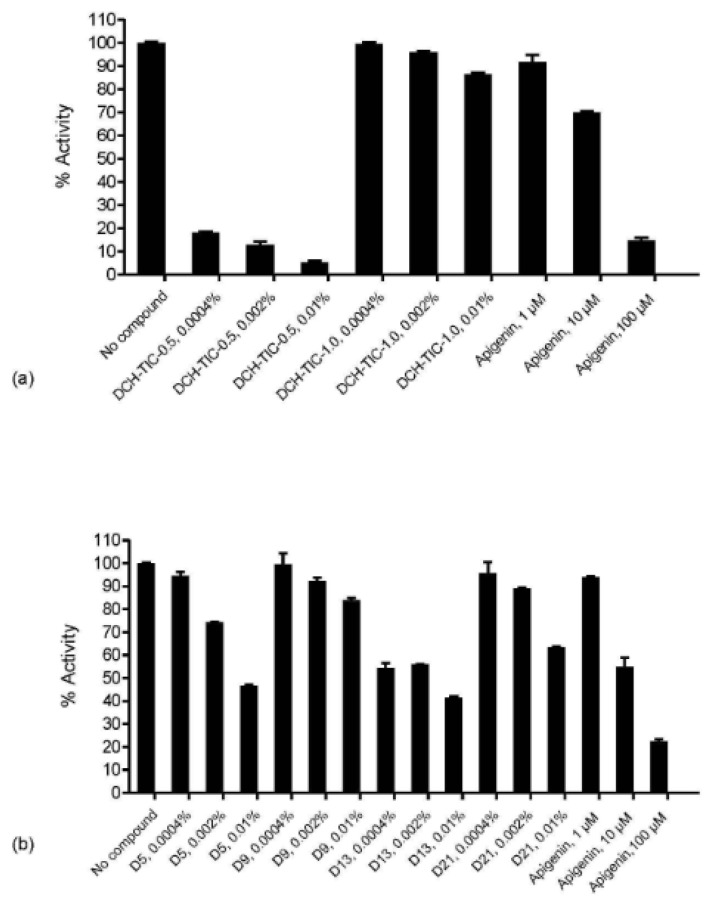
Cluster of Differentiation (CD) 38 hydrolase percent (%) activity following incubation with DailyColors^TM^. (**a**) DailyColors^TM^ (DCH-Total Ion Chromatogram (TIC)-0.5) exhibits strong dose-dependent inhibition, reducing activity of CD38 to 18 at 0.0004%, 13% at 0.002%, and 5% at 0.01%. Results for DailyColors^TM^ DCH-TIC-1.0 were comparable to those obtained for Apigenin. (**b**) DailyColors^TM^ (D5, D9, D13, D21) demonstrates comparable concentration-dependent results relative to Apigenin across all concentrations tested.

DailyColors™ demonstrated broad enzymatic and kinase-modulating activity across multiple in vitro assays relevant to aging, inflammation, and neurodegeneration. It significantly inhibited CD38 (Figure 2), a key enzyme associated with chronic disease and aging [34], with effects comparable to known reference inhibitors. Similar effects were observed for CD39 and CD73 activities (unpublished findings), which, along with CD38, are enzymes involved in NAD^+^ metabolism and chronic inflammatory signaling.

**Figure 3:**
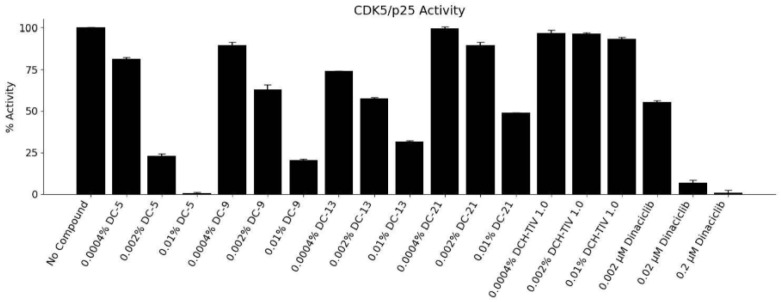
Cyclin-dependent kinase 5/p25 percent (%) activity following incubation with DailyColors^TM^ (DC). Concentrations of the MD-inspired dietary supplement (0.0004, 0.002, and 0.01%) (DC-5, DC-9, DC-13, DC-21, DC-31, and DCH-Total Ion Value (TIV) 1.0) were tested in duplicate in cyclin-dependent kinase 5/p25 (CDK5/p25), alongside pharmacological inhibitor Dinaciclib (10 µM). Lower concentrations (0.0004%) show minimal effect, while higher concentrations (0.002% and 0.01%) show reduced CDK5/p25 activity, with some formulations inducing CDK5/p25 inhibition below 30%. The reference inhibitor Dinaciclib exhibits potent inhibition at 0.2 μM.

The blend also strongly reduced CDK5/p25 activity, a pathway associated with neuronal dysfunction (Figure 3), and produced concentration-dependent inhibition of Jak1, Jak2, and Jak3, key mediators of cytokine-driven immune and inflammatory responses (unpublished findings). Elevated or dysregulated Jak activity is linked to autoimmune disorders, chronic inflammation, and certain cancers. Similarly, the MD-inspired dietary supplement demonstrated dose-dependent inhibition of recombinant human BACE1 activity in vitro, showing effects comparable to the reference inhibitor Verubecestat and suggesting a potential to reduce β-amyloid formation (unpublished findings). These findings suggest that the MD-inspired dietary supplement may modulate neuronal signaling pathways, potentially attenuating phosphorylation events associated with neurodegenerative processes and inflammation.

**Figure 4:**
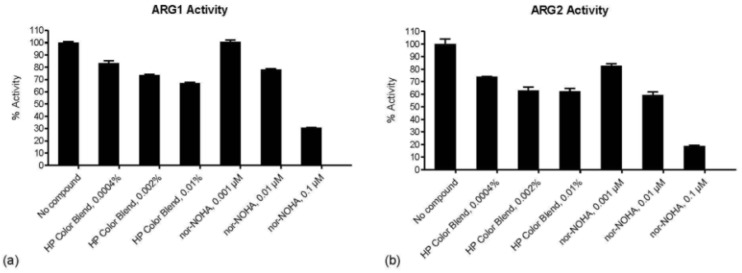
Arginase type 1 (ARG1) and ARG2 percent (%) activity following incubation with DailyColors^TM^. (**a**) DailyColors^TM^ Blend (HP Color Blend) demonstrates a concentration-dependent reduction, with activity decreasing from 83% at 0.0004% to 67% at 0.01%. The untreated control maintains 100% ARG1 activity. N^ω^-hydroxy-nor-L-arginine (Nor-NOHA, reference inhibitor) exhibits similar inhibition, reducing activity from 100% at 0.001 µM to 31% at 0.1 µM. (**b**) ARG2 activity decreases from 74% at 0.0004% to 62% at 0.01% DailyColors^TM^ and 83% at 0.001 µM to 19% at 0.1 µM Nor-NOHA.

In an arginase inhibitor assay, DailyColors™ further inhibited arginases type 1 (ARG1) and type 2 (ARG2), which are frequently upregulated in various human pathologies and represent critical targets for the development of anti-aging therapeutics. Concentration-dependent decreases in ARG1 and ARG2 activity were recorded following exposure to the MD-inspired dietary supplement, with the effect more prominent in ARG1 activity (Figure 4). The potency of inhibition was similar to the reference inhibitor N^ω^-hydroxy-nor-L-arginine (Nor NOHA).

**Figure 5:**
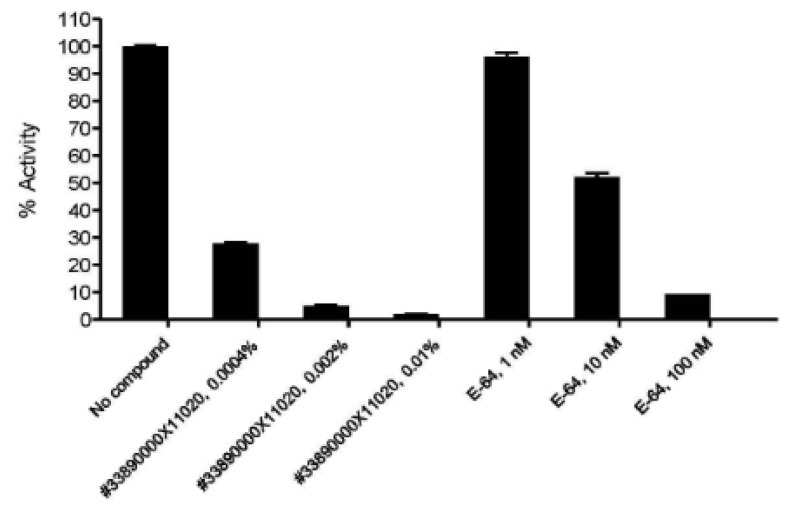
Cathepsin S percent (%) activity following incubation with DailyColors^TM^. Cathepsin S activity was observed at 28%, 5%, and 2% following treatment with 0.0004%, 0.002%, and 0.01% DailyColors™ powder blend (#33890000X11020), respectively. Comparable inhibition was observed with 1, 10, and 100 nm of E-64 (control inhibitor), where activity was recorded at 96%, 52%, and 9%, respectively.

Furthermore, DailyColors^TM^ showed strong, dose-dependent suppression of Cathepsin S, a protease central to antigen presentation and immune activation, achieving inhibition at lower concentrations than the control inhibitor E 64 (Figure 5). The inhibition of Cathepsin S demonstrates the MD-inspired dietary supplements’ modulatory effect on immune regulation in vitro. DailyColors™ also showed concentration-dependent inhibition of Keap1–Nrf2 binding in vitro, similar to the reference compound (ML334), indicating potential antioxidant activity (unpublished findings).

Collectively, in vitro screening using a battery of studies that focused on enzyme inhibition showed that DailyColors™ inhibits several enzymes and kinases involved in aging, inflammation, antioxidant properties and neurodegenerative pathways. The MD-inspired dietary supplement significantly inhibited activity of a broad array of specific enzymes, including CD38, CD39, and CD73; enzymes tied to NAD^+^ metabolism and chronic inflammation; and suppressed hyperactive CDK5/p25 and Jak1/2/3 signaling, which are linked to neuronal dysfunction and inflammatory disorders. DailyColors™ also inhibited ARG1/ARG2, enzymes associated with age-related metabolic decline, and strongly suppressed Cathepsin S, a regulator of immune activation. Together, these findings suggest that DailyColors™ broadly modulates biological pathways relevant to healthy aging, immune balance, and neuroprotection, and provide mechanistic insights into how the MD modulates healthy aging.

#### 3.1.2. Impact of MD Constituents on Cellular Models

Healthy aging and longevity are fundamentally rooted in maintaining robust cellular function, making cellular health a critical focus in understanding the MD-driven benefits. Because the MD influences multiple interconnected biological pathways, examining diverse cellular models provides meaningful insight into the MD’s systems-biology mechanisms. In this context, DailyColors™ evaluated a broad set of key cellular health pathways, spanning mitochondrial antioxidant effects, glucose sensitivity, cytokine inhibition, and gene expression related to cellular senescence, among other targets.

#### 3.1.3. Inflammation and Oxidation in Primary Human Monocytes

**Figure 6:**
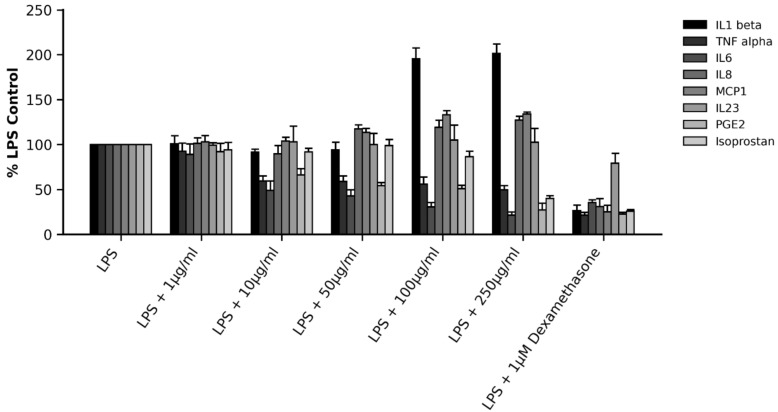
Effect of DailyColors^TM^ on inflammatory parameters in primary human monocytes. DailyColors^TM^ reduced LPS-induced interleukin (IL)-6, Tumor Necrosis Factor-alpha (TNFα), and Prostaglandin E_2_ (PGE_2_) secretion at ≥10 µg/mL, while isoprostane suppression was more prominent at higher doses. IL-23 was unaffected, whereas IL-8 and monocyte chemoattractant protein-1 (MCP-1) slightly increased, and IL-1β markedly increased. Dexamethasone strongly inhibited all markers, with the exception of IL-23, which only demonstrated slight inhibition.

The MD-inspired supplement showed strong anti-inflammatory activity in primary human monocytes (Figure 6). DailyColors^TM^ extract significantly suppressed lipopolysaccharide (LPS)-driven secretion of interleukin (IL)-6, Tumor Necrosis Factor-alpha (TNFα), and Prostaglandin E_2_ (PGE_2_), with inhibition evident even at the lowest tested concentration of 10 µg/mL. Isoprostane decreased only at higher concentrations, while IL-23 was unaffected, and IL-8, monocyte chemoattractant protein-1 MCP-1, and IL-1β increased slightly. Collectively, these findings indicate that this MD-inspired supplement exerts pronounced anti-inflammatory effects in primary human monocytes, particularly through targeting IL-6, TNFα, and PGE_2_, which are key mediators implicated in chronic inflammatory disorders.

#### 3.1.4. Anti-Inflammatory, Antioxidant and Neuroprotective Pathways

**Figure 7:**
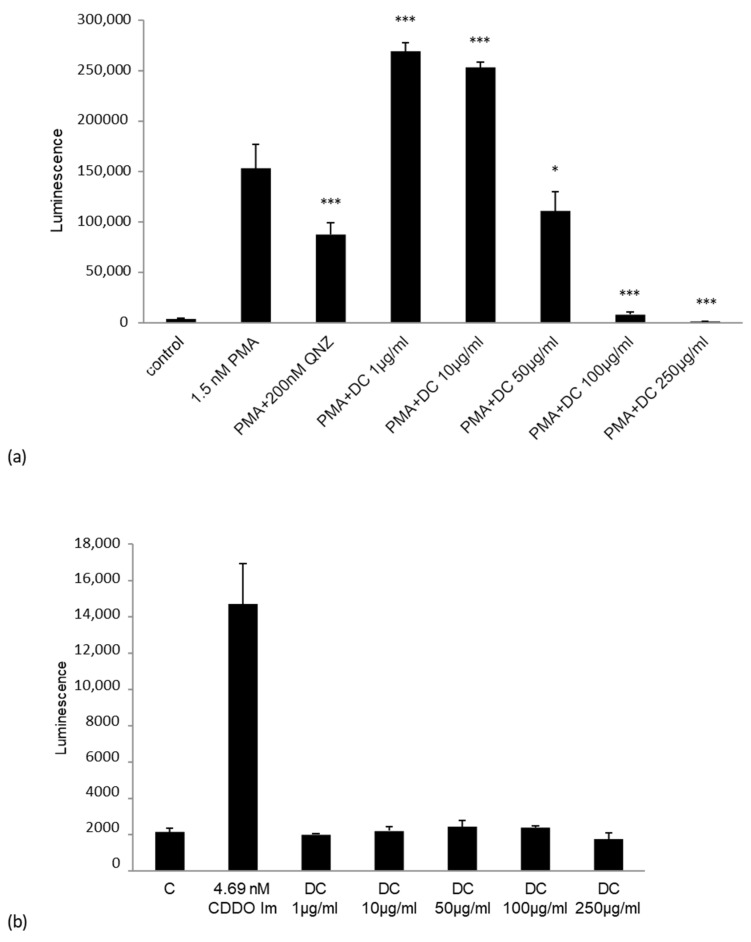
Effect of DailyColors^TM^ treatment on nuclear factor kappa-light-chain-enhancer of activated B cells (NF-κB) and phorbol 12-myristate 13-acetate (PMA)-induced nuclear factor (erythroid-derived 2)-like 2 (Nrf2). (**a**) At lower concentrations of DailyColors^TM^ (1–10 µg/mL), PMA-induced NF-κB activation showed a slight increase. Doses of 50 µg/mL and above produced significant progressive inhibition (* *p* < 0.05), with near-complete suppression at 100–250 µg/mL (*** *p* < 0.001). This effect surpassed that of the standard inhibitor QNZ. (**b**) No effect was found on Nrf2 activation (*p* > 0.05). * *p* < 0.05, and *** *p* < 0.001.

Nuclear Factor (erythroid-derived 2)-like 2 (Nrf2) and nuclear factor kappa-light-chain-enhancer of activated B cells (NF-κB) activation were evaluated in HEK293T cells to investigate the effect of DailyColors^TM^ as an antioxidant and anti-inflammatory regulator (Figure 7). Phorbol 12-myristate 13-acetate (PMA)-induced NF-κB activation was slightly enhanced at low extract doses (1–10 µg/mL), while higher doses (≥50 µg/mL; *p* < 0.05) progressively inhibited activation, with near-complete suppression at 100–250 µg/mL (*p* < 0.001) surpassing the known inhibitor QNZ, indicating significant anti-inflammatory potential. Similarly, in RAW 264.7 mouse macrophages, DailyColors™ blocked H_2_O_2_-induced reactive oxygen species (ROS) production at all tested doses, supporting robust antioxidant activity (unpublished findings).

In a different assay, neuroprotective activity was assessed by acetylcholinesterase activity using the Amplite^TM^ Colorimetric assay (AAT Bioquest, Pleasanton, CA, USA) following exposure to DailyColors^TM^. The MD-inspired supplement substantially inhibited acetylcholinesterase (AChE), resulting in elevated acetylcholine availability (unpublished findings). Elevated acetylcholine availability enhances neurotransmission in cholinergic pathways, thereby improving cognitive function and neuromuscular transmission.

#### 3.1.5. Metabolic Effects

**Figure 8:**
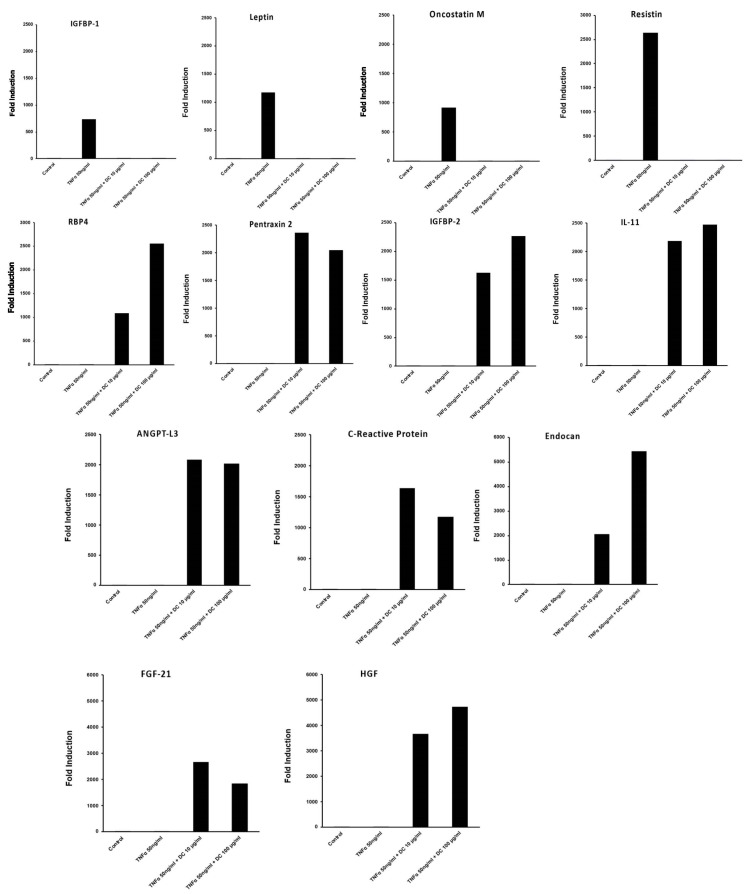
Effect of DailyColors^TM^ on Tumor Necrosis Factor-alpha (TNF-α)-induced secretion of pro-inflammatory adipokines. DailyColors™ (DC) suppressed TNF-α-induced secretion of pro-inflammatory adipokines (insulin-like growth factor-binding protein (IGFBP)-1, leptin, oncostatin M, resistin) in 3T3-L1 cells. Co-treatment with TNF-α and DailyColors™ upregulated beneficial metabolic and signaling proteins (angiopoietin-like protein 3 (ANGPT-L3), C-reactive protein, Endocan, fibroblast growth factor (FGF-21), hepatocyte growth factor (HGF), IGFBP-2, interleukin (IL)-11, retinol-binding protein 4 (RbP4), Pentraxin 2, which were not induced by TNF-α alone.

The evaluation of DailyColors^TM^’s effect on the secretion of adipokines associated with obesity and metabolic disease was conducted in differentiated 3T3-L1 cells (Figure 8). The MD-inspired dietary supplement fully inhibited TNF-α-induced secretion of pro-inflammatory adipokines (insulin-like growth factor-binding protein (IGFBP)1, leptin, oncostatin M, resistin), indicating strong anti-inflammatory activity. Conversely, beneficial metabolic proteins such as angiopoietin-like protein 3 (ANGPT-L3), C-reactive protein (CRP), Endocan, fibroblast growth factor (FGF)-21, hepatocyte growth factor (HGF), IGFBP-2, IL-11, retinol-binding protein 4 (RbP4), and Pentraxin 2 were upregulated with the co-treatment of TNFα and the MD-inspired dietary supplement, which were not induced by TNFα alone, suggesting that the MD-inspired formulation actively promotes the synthesis of these metabolic and signaling proteins. In another study, the MD-inspired dietary supplement demonstrated greater insulin-sensitizing effects in a C2C12 mouse myoblast cell line compared to the antidiabetic agent, rosiglitazone (unpublished findings).

Altogether, the experiments demonstrate that DailyColors™ has broad cellular benefits across inflammatory, oxidative stress, neuroprotective, and metabolic-related pathways. In primary human monocytes, the extract strongly inhibited LPS-induced IL-6, TNFα, and PGE_2_, even at low concentrations, indicating potent anti-inflammatory activity, while other markers showed minimal or dose-specific modulation. The MD-inspired dietary supplement also reduced NF-κB activation at higher concentrations and suppressed ROS generation in macrophages, supporting the formulations significant antioxidant potential. Neuroprotective effects were observed through reversible inhibition of AChE, allowing for greater acetylcholine availability and enhanced muscarinic and nicotinic cholinergic neurotransmission. Central cholinergic transmission is critical in learning, memory, and neurodegenerative diseases associated with aging.

#### 3.1.6. Anti-Aging

*Caenorhabditis elegans* is widely used as a primary research model in longevity science due to its short lifespan, well-mapped genetics, and highly conserved cellular pathways, which make it ideal for studying mechanisms of aging and health span [35]. Its reproducibility and well-established characteristics make *C. elegans* a powerful system for investigating how nutritional compounds, such as DailyColors™, influence cellular resilience and longevity-related pathways.

In human dermal fibroblasts, the MD-inspired dietary supplement downregulated aging-related markers (*p16INK*, *p21*, *c-FOS*, and *HGF*), indicating broad suppression of genes involved in cell cycle regulation and growth signaling (unpublished findings). It also modulated Advanced Glycation End Products (*AGE*) gene expression in a dose-dependent manner, suggesting reduced *AGE*-related oxidative and inflammatory stress.

**Figure 9:**
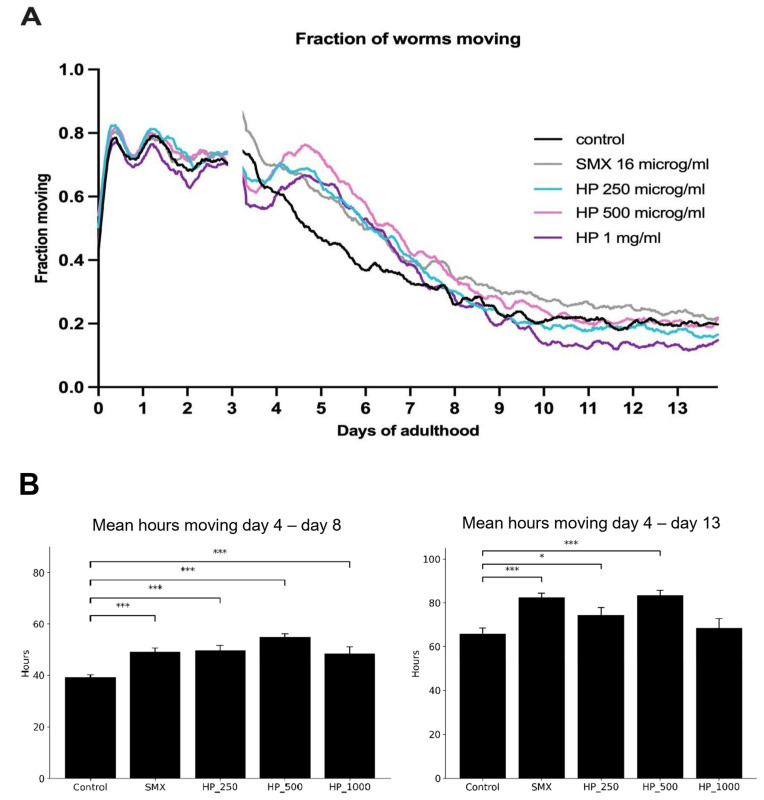
Effect of DailyColors^TM^ on *C. elegans* mobility in adulthood following exposure from early middle age onwards. (**A**) DailyColors™ (HP) doses significantly increased movement compared to control, with 500 µg/mL showing the strongest effect, from days 4 to 8. (**B**) A significant dose-dependent increase in distance moved was recorded, with 500 µg/mL producing the greatest effect from days 4 to 8. Over days 4 to 13, 500 µg/mL remained the most effective concentration (*** = *p* < 0.002, * = *p* < 0.05). Sulfamethoxazole (SMX) was used as the standard comparator.

**Figure 10:**
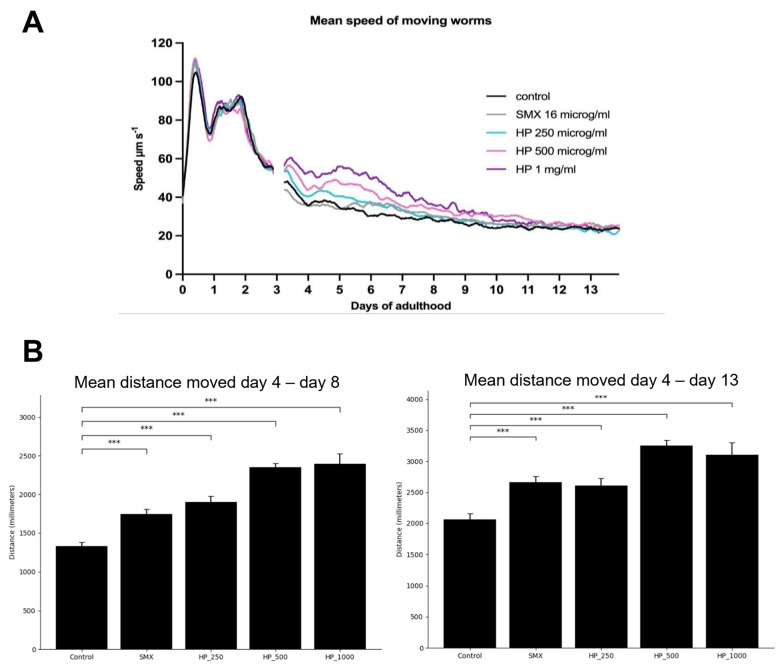
Effect of DailyColors^TM^ on mean *C. elegans* speed and mean distance moved in adulthood following exposure from early middle age onwards. (**A**) Worms displayed a clear dose-dependent increase in distance traveled. (**B**) All concentrations of DailyColors^TM^ (HP) produced statistically significant increases in mean distance moved, with 500 µg/mL producing the highest effect from days 4 to 8 compared to the control. (**B**) Across the full observation period (days 4 to 13), 500 µg/mL remained the most effective concentration overall (*** = *p* < 0.002). Sulfamethoxazole (SMX) was used as the standard comparator.

Using a different model, a Health Span assay was conducted to evaluate the age-slowing effect of DailyColors^TM^ formulations in an early middle-aged *C. elegans* model. The Health Span assessment endpoints included the fraction of worms moving with time throughout the experiment (Figure 9) and the mean speed of all worms (Figure 10). Across concentrations (250, 500, and 1000 µg/mL), the supplement improved worm mobility in a concentration-dependent manner. The 500 µg/mL concentration produced the strongest and most sustained improvement, exceeding both the control and the standard comparator (sulfamethoxazole, SMX, *p* < 0.01) and maintaining significant benefits through day 13. Over the extended period from day 4 to day 13, significant improvements persisted for 250 µg/mL, 500 µg/mL, and SMX, whereas the 1 mg/mL dose did not maintain a significant effect. Speed analysis showed a similar dose-dependent improvement from days 4–8, where worms showed a statistically significant dose-dependent increase in distance moved from that observed in the control when comparing 250 µg/mL and 500 µg/mL. Similar findings were observed in an earlier study conducted using the *C. elegans* model in adulthood (unpublished findings). Therefore, the findings suggest that the MD-inspired dietary supplement can slow aging and improve health in *C. elegans*, which is in line with evidence demonstrating the beneficial effects of the MD on longevity.

The findings from the various studies conducted highlight that the DailyColors™ supplement exerts multiple beneficial effects across diverse cellular models. The MD-inspired supplement enhances glucose uptake, modulates key genes associated with aging and cellular senescence, and suppresses key mediators of oxidative and inflammatory stress. These multi-pathway actions mirror protective effects observed in populations adopting an MD. Collectively, the results support the potential of the MD-inspired dietary supplement as a health-promoting nutraceutical for mitigating age-related processes and contributing to the prevention or management of various human diseases.

### 3.2. Effect of MD Constituents on Cognitive Health and Healthy Aging in Human Clinical Trials

Following preclinical investigations into enzyme inhibition, cellular mechanisms and effects in the *C. elegans* model, human clinical trials became the focus for the demonstration of efficacy. Two randomized, double blind, placebo-controlled trials assessed the synergistic effects of the polyphenols, building on the efficacy previously shown for individual plant-based components (Section 3.1 and Section 4).

Chong et al. [36] conducted a randomized, double-blind, placebo-controlled, cross-over trial wherein the effects of the DailyColors^TM^ blend were assessed on blood biomarkers associated with diverse mechanisms implicated in aging and age-related diseases, including mitochondrial function, inflammation, and oxidative stress, as well as on saliva DNA methylation patterns [36]. Thirty adult participants with a body mass index (BMI) over 25 were recruited into this clinical trial that involved a one-week-long treatment period, separated by a one-week washout period. The participants received either a placebo or DailyColors^TM^ at 150 mg/day. Following the cross-over intervention, participants were invited to a one-month open-label study to better understand chronic, daily consumption for a longer duration. Consenting participants were to consume the MD-inspired dietary supplement once daily for an extra month, starting 3 to 8 weeks after the cross-over intervention ended.

During the placebo period, there was a significant increase in blood CD38 concentrations from baseline to 24 h (*p* = 0.019), which was not observed in the active period [36]. There was also a decreasing trend of plasma 4-hydroxynonenal (4-HNE) levels after only one week of supplementation with DailyColors™. Increased CD38 has been reported to be associated with mitochondrial dysfunction and inflammation [37], while 4-HNE is an oxidative stress biomarker [38]. Beyond NAD^+^ metabolism, CD38 is a central regulator of immune signaling, calcium flux, and inflammatory cascades that become chronically dysregulated with age. Therefore, its inhibition helps rebalance immune function, dampen inflammaging, and stabilize cellular communication networks that drive tissue decline and age-related disease. Thus, the MD’s phytochemicals, including polyphenols, modulate immune signaling, oxidative stress, and inflammatory enzymes, indirectly lower CD38 activity and help maintain balanced cellular signaling that supports healthy aging. Collectively, the results suggest that supplementation with the MD-inspired dietary supplement was associated with potential occasional anti-inflammatory effects, improved mitochondrial function and reduced overall oxidative stress. Furthermore, a one-month open-label follow-up in 26 participants showed hypermethylation of the candidate CpG site cg13108341 (q-value = 0.021), which is the opposite trend observed over time at this site in those with advancing age [36]. DNA hypermethylation is a strategy used by the cell to mute areas of problematic gene expression and stabilize repetitive DNA coding regions from subsequent damage. Therefore, the results suggest that DailyColors™ supplementation may be beneficial to health by modifying gene regions targeted for age-related changes.

In a second study, DailyColors^TM^ was investigated in a 60-day, double-blind, placebo-controlled, randomized trial in 150 UK adults aged 50+ with a BMI ≥ 25 to investigate the effects on cognitive health and physical fitness [12]. Participants received either a medium (750 mg, n = 50) or high (2000 mg, n = 51) dose of DailyColors™ (~300 mg and ~750 mg polyphenols, respectively), or a placebo (n = 51). A sub-group (n = 15 per group) underwent additional assessments, including blood pressure measurements, characterization of circulating EVs and tandem-mass-tagged serum proteomics. Both doses improved cognitive performance, while the high dose enhanced reaction time and physical fitness measures. Proteomic profiling revealed significant modulation of pathways linked to inflammation, immunity, vesicle-mediated transport, and high-density lipoprotein (HDL) assembly. Proteomic analysis showed that supplementation with the MD-inspired dietary supplement significantly downregulated proteins involved in both adaptive and innate immune pathways, as expressed by significantly reduced serum protein expression in immune and pre-β1-HDL pathways, suggesting anti-inflammatory effects. Pre-β1-HDL proteins are typically elevated in obesity [39]; their reduction suggests a reversal of this obesity-related effect. The observed downregulation of these proteins represents ‘normalization’ of a known obesity-induced phenomenon. These results are consistent with widespread findings in humans that suggest that the MD increases HDL and improves indices of HDL quality [40,41,42]. Collectively, the findings suggest a trend for supplementation with the MD-inspired dietary supplement to enhance cognitive function and physical fitness, with dose-dependent effects evident for cognitive measures and physical fitness outcomes, in addition to improving systemic health in older, overweight adults. These improvements and the changes in immune-related and HDL protein pathways are consistent with the well-documented effects of the MD. Thus, the MD-inspired dietary supplement mimics key MD effects over time, suggesting it could offer an accessible way to support early-stage cognitive function in the aging population.

The biological, functional, and omics signals observed in the two DailyColors^TM^ clinical studies are in accordance with the established clinical literature on the MD, which consistently demonstrates benefits across cardiometabolic, inflammatory, immune, and cognitive aging domains. For example, a higher intake of polyphenol-rich plant foods, as shown in the Nurses’ Health Observational Study (n = 121,700), was linked to healthier aging outcomes, including lower frailty, slower cognitive decline, and reduced mortality risk [43,44,45]. DailyColors^TM^ clinical trials similarly show complementary interventional data suggesting coordinated functional, proteomic, and epigenetic effects consistent with a systems biology mechanism characteristic of MD plant intake rather than isolated nutrient effects. Furthermore, in the PREDIMED randomized controlled trial (RCT), a study that assessed the MD’s effects on the risk of major cardiovascular events, sustained consumption of the MD reduced cardiometabolic risk, inflammation, and cognitive decline, with mechanistic sub-analyses identifying dietary polyphenols as key biological drivers [46]. Thus, the MD-inspired dietary supplement studies align with these findings by demonstrating that diverse, concentrated MD-derived polyphenols can simultaneously modulate multiple aging-related biological pathways. Importantly, these effects are increasingly understood as emergent properties of a systems biology intervention, arising from the coordinated action of diverse, polyphenol-rich plant foods rather than isolated nutrients or single pathways. DailyColors^TM^ reflects this same systems-level biology by capturing the multi-compound, multi-pathway influence of MD plants, particularly extra-virgin olive oil, vegetables, legumes, fruits, herbs, and spices, through measurable shifts in proteomic and epigenetic networks relevant to healthy aging.

## 4. Summary on the Benefits of Mediterranean Diet Constituents for Cognitive Health, Antioxidant Potential and Metabolic Health

Extensive evidence shows that the MD supports broad health benefits through synergistic interactions among its plant-derived bioactive compounds. Research into supplementation with constituents from the MD reimagines the diet as a programmed routine intended to satisfy the molecular demands of a healthy biochemical network, rather than a simple dietary pattern. The polyphenolic components serve as the coded elements of the program to influence convergent pathways, including CD38-linked NAD^+^ metabolism, cytokine signaling, mitochondrial activity, and lipid transport—functioning as an integrated systems-biology intervention to counter competing programs that arise from senescence and disease. Because these effects arise from many interacting phytochemicals, including polyphenols, isolating the impact of any single ingredient is difficult. Accordingly, the following section highlights select constituents in both the MD and the MD-inspired dietary supplement, DailyColors™, with their demonstrated benefits in randomized clinical trials, summarized in Table 2.

### 4.1. Impact of Nutritional Components in the Mediterranean Diet on Cognitive Function

A substantial body of evidence links adherence to the MD with better cognitive performance [47]. This section highlights studies examining MD-related nutrients and the MD-inspired dietary supplement on cognition.

In a 6-month randomized, placebo-controlled trial, Whyte et al. [48] tested blueberry powders and a high-potency blueberry extract in older adults. Only the extract improved episodic memory, showed marginal visuospatial benefits at 3 months, and reduced systolic blood pressure, suggesting that concentrated blueberry compounds may be more effective than whole powder forms. Blueberries, in addition to other berries, are rich sources of anthocyanins [63]. A systematic review of 49 RCTs similarly found that berry anthocyanins improved memory, attention and psychomotor speed in individuals with mild cognitive impairment [49].

Complementing these single-ingredient studies, a 60-day RCT of the MD in a capsule showed significant cognitive benefits in adults aged 50+, with improved reaction time in the high-dose group and improved accuracy across both active groups. These findings align with MD-associated cognitive advantages and support the synergistic effects of its combined bioactive compounds [12]. In fact, similar cognitive benefits have been reported in studies assessing the MD, where adherence to the diet was linked to a slower rate of cognitive decline [64]. This effect may be partly driven by the diet’s ability to reduce systemic inflammation, a factor associated with lower all-cause dementia risk [51].

### 4.2. Antioxidant/Anti-Inflammatory Properties of the Mediterranean Diet

Beyond cognitive benefits, the MD is strongly linked to antioxidant activity due to the high content of antioxidants in fruits and vegetables, including polyphenols, vitamins, and carotenoids [50]. Clinical trials using tomato ingredients standardized to lycopene (4–20 mg/day) consistently showed reductions in oxidative stress markers such as malondialdehyde (MDA), TNF-α, and thiols over periods ranging from 2 weeks to 6 months (Table 2, [52,53,65]). A meta-analysis of green coffee bean extract, an MD-relevant source of the natural chlorogenic acid (CGA) polyphenol [66], found that supplementation (50–1200 mg/day for 8–12 weeks) reduced TNF-α, indicating lowered systemic inflammation [67].

Additional evidence comes from ginger, a source of gingerol and shogaol [68]. Trials in patients with knee osteoarthritis showed improvements in pain scores, quality of life, and knee circumference after ginger-based supplementation, suggesting potential anti-inflammatory and pain-reducing effects [69]. Systematic reviews further demonstrated significant increases in total antioxidant capacity, glutathione peroxidase activity, and reductions in MDA, CRP, and high-sensitivity CRP across studies using 1.4–3 g/day of ginger extract or powder [70,71,72]. While evidence for pain relief is mixed, results generally support ginger’s anti-inflammatory and antioxidant effects.

Together, these findings highlight the strong antioxidant and anti-inflammatory potential of MD components. These effects are driven by the abundance and synergy of phytoconstituents in the various MD-derived fruits and vegetables [50], benefits that can also be replicated and quantified using MD-inspired formulations, as will be discussed further below.

#### Antioxidant Potential Quantified by ORAC for the MD and DailyColors^TM^

The ORAC (Oxygen Radical Absorbance Capacity) value, or “ORAC score”, is an easy method for assessing the in vitro antioxidant capacity of different foods and supplements to negate the effects of free radical formation in cells [73], while in vivo antioxidant capacity is more difficult to perform. Foods high in ORAC values are postulated to better neutralize free radicals, aligning with the free radical theory of aging, which links excess oxidative damage to age-related decline [74,75]. Early USDA reports suggested that diets rich in high-ORAC fruits and vegetables, such as spinach and blueberries, may help slow aging and preserve memory and learning, observations now supported by evidence showing the MD’s strong antioxidant profile due to its abundance of antioxidants, including polyphenols, vitamins, and carotenoids [53].

Cellular and tissue damage can result from oxidative stress when ROS and reactive nitrogen species (RNS), including superoxide, nitric oxide, hydrogen peroxide, and peroxynitrite, damage lipids, proteins, and DNA, factors which ultimately can contribute toward the pathogenesis of various diseases [76,77]. However, an internal mitochondrial antioxidant network (e.g., glutathione peroxidase, manganese superoxide dismutase, ubiquinol, glutathione) works to neutralize free radicals by donating electrons, thereby preventing cellular injury [56,78]. Studies demonstrate this protective activity, such as glutathione peroxidase rapidly reducing H_2_O_2_ in cardiac mitochondria and mitochondrial reductants, quenching peroxynitrite formation in liver tissue [79,80].

Increasing antioxidant defenses can be accomplished through dietary intake of known antioxidant-rich foods with high ORAC values. ORAC quantifies antioxidant density, with suggested daily intake ranging from 3000 to 5000 ORAC units [81]. Spices and berries rank highest, while processed grains and animal products score lower. Foods can be grouped into tiers of ORAC values (Table 3), providing guidance for constructing antioxidant-rich diets.

As can be demonstrated in Table 4, DailyColors^TM^ (the MD in a capsule) has four ingredients (grape, ginger, European elder, and rosemary) in the first tier of ORAC ranking (ingredients with ORAC values over 10,000), two ingredients in the next tier (blueberry and black currant), and two ingredients in the lowest tier (pomegranate and apple). Thus, consumption of the MD-inspired dietary supplement and the MD can be beneficial for health by scavenging ROS and free radicals and protecting against cellular or mitochondrial oxidative damage/stress, in addition to supporting mitochondrial metabolic health, slowing healthy aging and providing memory support.

### 4.3. Effects of Components of the Mediterranean Diet on Metabolic Health

Metabolic syndrome (or syndrome X) is often mistakenly treated as a metabolic disease, but under the Food, Drug, and Cosmetic Act it is not a disease state, rather, it is a cluster of clinical markers that signal a higher risk to the clinician and, warrant closer monitoring for conditions like prediabetes or diabetes both of which are disease states under the Food, Drug, and Cosmetic Act [77,78]. Metabolic syndrome typically involves five risk factor areas: abdominal obesity as assessed by waist circumference, elevated blood pressure, impaired fasting glucose, high triglycerides, and low HDL cholesterol, which are all risk factors for the development of disease. As was summarized in Table 2, adherence to the MD has been linked to improvements in these metabolic parameters—including body weight, glucose regulation, blood pressure, and lipid profile. The following section highlights specific MD components shown to influence these outcomes. Foods can be assessed by the US FDA for their ability to reduce the risk of developing disease when data is submitted as part of a health or qualified health claim in a Citizen Petition.

#### 4.3.1. MD Components and Body Weight

Several MD plant components have been studied for their effects on body weight and obesity related outcomes. A meta-analysis of 14 RCTs found that 0.5–1 g/day of ginger extract over 2–12 weeks significantly reduced body weight (Standardized Mean Difference, SMD, −0.66; 95% CI, −1.31, −0.01; *p* = 0.04) and waist to hip ratio (SMD—0.49; 95% CI, −0.82, −0.17; *p* = 0.003), though not BMI (standardized mean difference (SMD)—0.65; 95% confidence interval (CI), −1.36, 0.06; *p* = 0.074), in overweight and obese participants [83]. Green coffee bean extract, rich in CGA polyphenols [66], also showed weight-reducing effects. A meta-analysis of six RCTs (46–1050 mg/day for 4–22 weeks) reported significant weight loss in overweight and obese individuals [84], with other trials in metabolic syndrome showing reductions in body weight, waist circumference, and BMI at CGA doses of 15–28 mg/day [85,86]. Other MD foods, such as tomatoes, produced similar effects, as daily tomato juice (32.5 mg lycopene) intake reduced waist circumference in healthy young women after two months [87].

Collectively, ingredients like ginger, CGA-containing plants, artichoke, and tomatoes can support weight management. These benefits are also observed when the synergism of various foods in the MD is combined. For example, an 18-month trial showed greater weight loss from an MD than from general dietary advice alone [54], and a meta-analysis confirmed significant improvements in BMI and waist circumference in those consuming the MD relative to other therapeutic strategies for metabolic syndrome [57]. Overall, while individual MD foods exert measurable benefits, it is more likely that all nutritional components of the MD work together synergistically to promote their beneficial health effects on a greater scale.

#### 4.3.2. MD Components and Blood Pressure

Green coffee bean extract, a rich source of CGA’s, has also been shown to modulate blood pressure [66]. For example, in an RCT conducted by Kozuma et al. [55], doses of 46–185 mg/day (providing 25–100 mg CGAs) over 28 days significantly reduced systolic and diastolic blood pressure in men with mild hypertension in a dose-dependent pattern. A meta-analysis of nine RCTs (501 participants; 46–800 mg/day for 4–16 weeks) further confirmed significant reductions in both measures, with the strongest effects seen in groups with hypertension or metabolic syndrome and at ≥400 mg/day green coffee bean extract for systolic blood pressure [88].

Similar benefits have been reported for berry anthocyanins. For example, across 22 RCTs, doses ranging from 1.35 to 724 mg/day improved vascular function and blood pressure [49]. Likewise, olive oil intake has been shown to be inversely associated with both systolic and diastolic blood pressure [89], likely through polyphenol-induced increases in nitric oxide and other vasoprotective mechanisms [58,59].

Collectively, CGA-containing plants, berries, and olive oil, which are all key MD components, support blood pressure regulation. These effects are also observed when foods are combined within the MD, as shown in a large, multicenter 4.8-year randomized trial [5] and another meta-analysis in adults with and without hypertension [60], which both demonstrated significant blood pressure reductions with incorporation of the MD. Supplementation with an MD-inspired dietary supplement has also shown systolic blood pressure modulation as demonstrated by a significant condition effect for systolic blood pressure, with a greater increase in systolic blood pressure in the high versus low dose condition after 60 days of supplementation [12]. Thus, there is evidence that both the individual components of the MD and all nutritional components of the MD combined, working together synergistically (e.g., the diet and MD-inspired supplements), can promote reductions in blood pressure.

#### 4.3.3. MD Components and Blood Glucose Control/Glycemic Responses

Regulating blood glucose is essential for maintaining metabolic balance, and the MD supports this through high-fiber, low-glycemic foods and healthy fats that slow sugar absorption and improve insulin sensitivity, and by modulation through anti-inflammatory phytochemicals, including polyphenols [90]. Clinical evidence shows that several MD components can help modulate glycemic control. In a 4-week RCT, polyphenols from pomegranate juice, but not extract capsules, reduced postprandial blood glucose in healthy adults [91]. CGA-rich green coffee bean extract has also shown benefit: Patti et al. [85] reported reduced fasting glucose in metabolic syndrome patients after 16 weeks of 28 mg/day CGA, and Castellino et al. [86] observed decreases in hemoglobin A1c (HbA1c) and plasma lipids after 24 weeks of 15–18 mg/day CGAs from artichoke extract. Ginger supplementation similarly lowered fasting glucose by up to 20% in peritoneal dialysis patients [92], and meta-analysis results of 14 RCTs show consistent fasting glucose-lowering effects (SMD −0.68; 95% CI −1.23, −0.05; *p* = 0.03) across overweight and obese populations [83]. Administration of an apple peel extract (350 mg/day, total phenolic content of 2.85 mg) also reduced blood sugar and HbA1c in hyperlipidemic women [93].

Overall, evidence indicates that CGA-containing plants, pomegranate, artichoke, ginger, and apple extracts can support glycemic control. The effects can also be observed when the synergism of various foods in the MD is combined, as adherence to the MD has been shown to be associated with improved fasting glucose homeostasis, insulin levels [90] and insulin resistance (e.g., Homeostatic Model Assessment of Insulin Resistance (HOMA IR)), supporting the idea that it is likely that all nutritional components of the MD work together synergistically to modulate blood glucose levels, rather than a single bioactive [57,90,94].

#### 4.3.4. MD Components and Triglycerides and Cholesterol

Cardiovascular diseases are the leading global cause of mortality, making prevention essential [95]. Evidence shows the MD supports cardiovascular health by improving lipid profiles. For example, consumption of an olive fruit extract containing hydroxytyrosol significantly lowered LDL-C in hypercholesterolemic patients [96]. These findings are corroborated by supplementation with onions and green coffee beans [97,98]. Both meta-analyses demonstrated significant improvements in lipid profiles (total cholesterol, HDL, and LDL) across diverse populations. Additional studies showed that CGA’s from artichoke extract reduced plasma lipids in metabolic syndrome [86], and ginger supplementation increased HDL, though there were no effects on triglycerides, or total- and LDL-cholesterol levels [83]. Berry anthocyanins were shown to improve vascular function, especially flow-mediated dilation, in another systematic review of 22 RCTs [49]. However, effects on cholesterol and triglycerides were inconsistent and varied by study design (different intervention durations, different test item composition), and the population, including physical activity levels.

Overall, MD components such as olives, onions, berries, ginger, and CGA-rich plants support healthier lipid profiles. Furthermore, the effects can be observed when the synergism of various foods in the MD is combined. A 4.8-year randomized trial showed higher HDL levels with increased polyphenol intake [5], and a meta-analysis confirmed MD-associated improvements in triglycerides and other lipid parameters [57]. Similarly, supplementation with the MD in a capsule produced proteomic shifts linked to improved HDL assembly and reversal of obesity related lipid abnormalities [39]. This result is consistent with widespread findings in humans that suggest that the MD increases HDL and improves indices of HDL quality [40,41,42]. Together, evidence shows that both individual MD foods and their synergistic combination can promote healthier blood lipid profiles.

In conclusion, across metabolic outcomes, evidence consistently indicates that individual MD components exert selective and often modest effects, whereas broader and more coherent benefits emerge when these components are consumed together as part of the MD pattern. Weight regulation, blood pressure, and glycemic control show the greatest consistency across individual foods and extracts, with CGA-rich plants, ginger, berries, olive oil, tomatoes, pomegranate, and apples reproducibly improving at least one metabolic marker, suggesting shared actions on insulin sensitivity, inflammation, vascular function, and mitochondrial metabolism. In contrast, lipid outcomes, particularly triglycerides and LDL cholesterol, are more variable when assessed in isolation and appear highly dependent on dose, duration, population characteristics, and baseline metabolic status, with more reliable effects observed for HDL quantity and quality. Notably, inconsistencies seen in single-component studies largely resolve at the dietary-pattern level, meaning that long-term MD trials and MD-inspired supplement studies demonstrate convergent improvements across weight, glucose homeostasis, blood pressure, and lipid metabolism, accompanied by favorable proteomic and epigenetic changes. Together, these findings support a central conclusion that the MD’s metabolic benefits arise not from any single bioactive, but from the synergistic, multi-pathway interactions of diverse polyphenols and nutrients consumed together, underscoring the importance of dietary complexity and systems-level integration for promoting metabolic health, longevity, and healthy aging.

### 4.4. Influence of Polyphenols on Health

Many of the MD’s health benefits stem from its rich polyphenol content, particularly anthocyanins. Epidemiological evidence suggests that these polyphenols are strongly linked to a reduced risk of chronic disease, healthy aging, and longevity [99]. Modern tools such as AI, metabolomics, and proteomics have further clarified how individual phytochemicals act on specific metabolic pathways [99]. Although few systematic reviews report precise dose–response thresholds, several analyses offer guidance.

Two systematic reviews on cognitive outcomes indicate that acute cognitive benefits generally require ≥250–300 mg of polyphenols, with higher doses needed for reaction time and markers like brain-derived neurotrophic factor; chronic benefits typically emerge at >500 mg/day or with high-bioavailability polyphenols [100]. A separate review recommends ≥ 138 mg/day of anthocyanins for at least 5 weeks to improve verbal memory and executive function [101]. Polyphenols also support athletic performance. Systematic reviews report performance improvements of ~2% with ≥7 days of supplementation, with effective doses averaging ~430–700 mg/day, though optimal levels remain uncertain [102,103].

As was described previously, strong evidence shows that the MD’s metabolic benefits are largely driven by its high polyphenol content. Systematic reviews suggest polyphenols can improve key features of metabolic syndrome, particularly at higher intakes (~500 mg/day) [104], though specific foods influence different outcomes. For example, tea improves anthropometrics; cocoa affects blood pressure; green tea, citrus products/flavanones with quercetin and soy impact lipids; and cocoa or cinnamon improve glucose. Interestingly, the authors suggested that no single food affects all markers, underscoring that the MD’s synergistic combination of polyphenols, rather than isolated ingredients, is responsible for the broad metabolic effects. This aligns with findings from MD-focused clinical trials and studies assessing MD-inspired dietary supplement formulations. Another systematic review emphasized that health outcomes are better predicted by overall dietary patterns than by single polyphenol compounds, though the main dietary products providing polyphenols were reported as tea, coffee, red wine, fruits, and vegetables, all of which are strongly associated with reduced chronic disease risk [105]. Importantly, these are all foods that can be found in the MD and MD-inspired dietary supplements, as has been discussed in detail throughout this review. Despite heterogeneity across studies (with respect to the selected population investigated, markers/endpoints measured, dietary habits and polyphenol food sources), this systematic review reported an estimation of the mean total polyphenol intake at 900 mg polyphenols/day. Furthermore, a higher total flavonoid intake (>500 mg/day) showed inverse associations with cardiovascular events, though exact optimal doses remain unclear [105]. While an optimal reference intake level for total polyphenols has yet to be determined, it was concluded that most data strongly support the protective effects of a polyphenol-rich dietary pattern.

Despite the clear importance of polyphenols, modern agricultural practices have reduced plant diversity and phytochemical availability [99]. The impacts of this loss are demonstrated by findings of a study reporting that the most important predictor of gut microbiome health (e.g., its diversity) is the diversity of plants consumed, rather than the quantity [106]. Given widespread reliance on processed foods and declining whole food phytochemical exposure, increasing phytochemical diversity is essential for health and longevity. While adopting the MD can achieve this, practical barriers, including cost, access, cultural factors, and dietary complexity, limit adherence [31]. Therefore, alternative approaches such as MD-inspired supplements can help deliver diverse phytochemicals in an accessible format.

## 5. Association Between MD Constituents and Biomarkers of Longevity

Biomarkers are measurable indicators of biological processes and are essential for tracking health, yet standardized aging biomarkers remain limited [107]. Nonetheless, emerging longevity biomarkers have been examined in the context of studies conducted on the MD and an MD-inspired dietary supplement. In a randomized, double-blind, placebo-controlled cross-over trial, Chong et al. [36] evaluated DailyColors™ in adults with BMI > 25, measuring blood markers associated with diverse mechanisms implicated in aging and age-related diseases, including mitochondrial function, inflammation, and oxidative stress, as well as on saliva DNA methylation patterns. Two recently developed biomarkers of senescence have been utilized: an epigenetic methylation pace clock and a MudhoAge clock to predict biological age, based on CpG methylation. After one week of supplementation followed by an open-label extension, study participants showed significant hypermethylation of cg13108341, a site typically hypomethylated with aging [108,109]. This shift suggests a reversal of an age-related epigenetic trend that may be due to the presence of high polyphenol levels that are associated with altered DNA methylation patterns and lower biological age [110,111]. This study also used the MuhdoAge saliva-based epigenetic clock, which is used to predict biological and memory age. This epigenetic clock has been validated for accurate biological age prediction with an R^2^ = 0.878 [112]. Preliminary findings show a dose-dependent reduction in “memory age” of 3.77 years after 60 days of DailyColors™ supplementation (Table 5).

Together, these biomarker changes indicate that both the MD and MD-inspired supplements may help reverse age-related signatures [108,109]. Given the MD’s high polyphenol content—long associated with favorable epigenetic patterns and reduced biological age—these effects are likely driven by its dense phytochemical (including polyphenol) profile, associated with altered DNA methylation patterns and a lower biological age [110,111].

## 6. Conclusions

**Figure 11:**
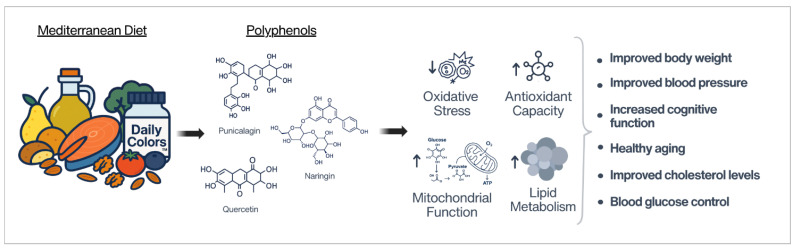
Human health benefits of the Mediterranean Diet (MD) and MD-inspired dietary supplementation. The MD, rich in colorful plant-based foods, provides polyphenols such as punicalagin, naringin, and quercetin, among many others. These compounds are known to reduce oxidative stress, enhance antioxidant capacity, support mitochondrial function, and regulate lipid metabolism. Collectively, these effects contribute to improved body weight, blood pressure, cholesterol levels, blood glucose control, cognitive function, and healthy aging. Beneficial effects can also be observed with MD-inspired supplementation, such as the DailyColors™ dietary supplement, delivering over 150 bioactive compounds derived from foods characteristic of the MD.

Extensive evidence shows that the MD supports healthy aging across multiple biological systems, largely due to its diverse phytochemicals, including polyphenols. Yet the precise mechanisms and synergistic interactions among these compounds remain only partly understood. This review summarizes research on individual MD components and on an MD-inspired dietary supplement, highlighting benefits across cognition, aging biomarkers, inflammation, oxidative stress, and metabolic health, all largely modulated by the MD’s high polyphenol content (Figure 11). The supplement was designed to capture the MD’s broad polyphenol diversity and showed multi-pathway effects in enzymatic assays, cellular models, and human trials, reflecting the network-level health and aging benefits seen in long-term MD adherence. No single food can improve all features of complex conditions like metabolic syndrome [104], reinforcing that the MD’s benefits arise from synergistic, multi-compound interactions. Thus, the investigation of a combined MD blend provided a unique opportunity to explore the broad polyphenol actions independent of whole-food variables such as fats, proteins, carbohydrates or fiber, revealing coordinated effects across multiple enzyme inhibition assays, proteomic pathways, cellular health pathways, and diverse pathways in the human studies, demonstrating improved cognitive function and physical performance, which are all effects that align with the epidemiological data from extensive MD research.

Studying single components of the diet in human clinical trials, while useful, brings only limited understanding as to the individual mechanisms behind the benefits of the MD, whereas studies of numerous constituents of the diet acting in concert, taken daily in a supplement, more closely approximate the entourage benefits of a maintained MD. Therefore, this review has demonstrated that the MD’s benefits emerge from interconnected pathways regulated by diverse polyphenols that regulate key enzymes of health and aging biology, a particularly important insight in an era of declining whole-food consumption. While full MD adherence can be challenging [31], MD-inspired supplementation as an approximate surrogate approach offers a practical approach to increasing polyphenol diversity and supporting health and longevity [12,36].

## Figures and Tables

**Table 1:** Components of the MD in a capsule (DailyColors^TM^ dietary supplement).

Active Blend Breakdown	Principal Polyphenol (s) Present	Dose/Day (mg)
Grape extract (*Vitis vinifera* fruit and leaves)		118.13
Ginger extract (*Zingiber officinale*, rhizomes)	Gingerol, Quercetin	75
European elder (*Sambucus nigra*, fruit)		18.23
Rosemary extract (*Salvia rosmarinus*, leaves)	Rosmarinic Acid	56.25
Blueberry extract (*Vaccinium caesariense*, fruit)	Anthocyanins	18.75
Black currant (*Ribes nigrum*, fruit)		18.23
Pomegranate extract (*Punica granatum*, fruit)	Punicalagin	75
Apple extract (*Malus pumila*, fruit)		75
Green coffee bean extract (*Coffea arabica*, seed)	Chlorogenic acids	56.25
Beet (*Beta vulgaris*, root)		18.38
Kale (*Brassica oleracea* var*. acephala*, leaf)		18.75
Grapefruit (*Citrus paradisi* extract, fruit)	Naringin	18.75
Olive extract (*Olea europaea*, fruit)	Hydroxytyrosol	47.85
Onion extract (*Allium cepa*, bulb)	Quercetin	75
Carrot (*Daucus carota sativus*, root)		18.38
Tomato (*Lycopersicon esculentum*, fruit)		13.88

**Table 2:** Summary of studies from peer-reviewed literature wherein ingredients and/or components within the Mediterranean Diet and DailyColors^TM^ were demonstrated to be efficacious in human populations.

Outcome(s)/Parameter(s) Tested	Duration of Use	Food and Dose/Serving Level	Population	Reference(s)
Cognitive Function				
Memory performance, cardiovascular function	1 h to 24 weeks	Blueberry juice/powders/extracts. Anthocyanin content of the intervention product was provided in all articles and ranged from 1.35 to 724 mg/day.	Healthy and patient populations suffering from metabolic syndrome, myocardial infarction, insulin resistance, or (pre-)hypertension.	[42]
Improved episodic memory performance in delayed word recognition	24 weeks	Wild blueberry extract at 500 mg and 1000 mg, and a purified extract at 100 mg.	Older adults with subjective mild cognitive impairment.	[40]
Antioxidant/Oxidative Stress				
Oxidative stress	2 weeks (2-week washout)	5, 10, 20 mg of lycopene from tomato ketchup or Lyc-O-Mato capsule per day	Healthy male and female subjects	[46]
Oxidative stress biomarkers, lipid profile (total cholesterol (TC), high-density lipoprotein (HDL), low-density lipoprotein (LDL))	6 months	4 mg lycopene/day	Healthy postmenopausal women	[47]
Inflammation, immunomodulation, oxidative stress	26 days	Lyc-o-Mato (5.7 mg of lycopene, 3.7 mg of phytoene, 2.7 mg of phytofluene, 1 mg of beta-carotene, and 1.8 mg of alpha-tocopherol)	Healthy men and women	[48]
Liver function and inflammatory biomarkers	8–12 weeks	50 to 1200 mg green coffee bean extract (GCBE)/day	Meta-analysis of healthy and disease populations (overweight/obese individuals with normal liver function and non-alcoholic fatty liver disease [NAFLD] adults)	[49]
Oxidative stress	4 to 12 weeks	1430 to 3000 mg/day of ginger extract powder; 1 to 3000 mg/day of ginger powder	Meta-analysis of studies conducted in patients with cancer, obesity, Type 2 diabetes (T2D), NAFLD	[50]
Inflammation and chronic pain	30 days	25 mg of ginger and 5 mg of *Echinacea*	Subjects with knee osteoarthritis	[51]
Pain	2 days to 6 months	500 mg to 12 g of ginger; 1 g of ginger powder; and 15 to 170 mg of ginger extract	Systematic review in disease conditions (e.g., osteoarthritis) and healthy participants (e.g., those undergoing acute eccentric exercise).	[52]
Inflammatory markers	4 to 12 weeks	1 to 3 g ginger/day	Meta-analysis of studies conducted in disease populations (e.g., T2D, NAFLD, osteoarthritis, breast cancer)	[53]
Metabolic Health				
Glycemic control/Blood glucose	4 weeks Acute for the juice	0, 200, or 400 mg of pomegranate extract (capsules providing 24 and 48 mg of punicalagin/day) or 12.4 mg of punicalagin/day (beverage)	Healthy adults	[54]
Glucose metabolism	45 days	350 mg/day of apple peel extract (providing 2.85 mg of total phenolic content)	Female population with hyperlipidemia	[55]
Body weight, BMI, waist circumference	16 weeks	28 mg chlorogenic acids (CGA)/day (provided from Kepar)	Adults with metabolic syndrome	[56]
Fasting glucose	10 weeks	1000 mg ginger	Peritoneal dialysis patients	[57]
Lipid profile (TC, LDL, and HDL)	2.5 months	25 mg of olive fruit extract/day (providing 5 mg hydroxytyrosol//day) in combination with red yeast rice (RYR)	Patients with and without statin-associated myalgia	[58]
Lipid profile (TC, LDL, HDL, and triglyceride levels)	2 to 12 weeks	46 to 800 mg of GCBE/day	Meta-analysis of studies conducted in healthy men, obese/overweight women, and patients with metabolic syndrome, hypertension, and NAFLD	[59]
Lipid profile (TC, LDL, HDL, and triglyceride levels)	2 to 12 weeks	150–200 mL onion extract/day, 100–1000 mg onion peel extract/day, 100 mL/day onion juice, and 900 mg/day steamed onion	Meta-analysis of studies conducted in patients with dyslipidemia	[60]
Blood pressure	4 to 16 weeks	46 to 800 mg of GCBE/day	Meta-analysis of studies conducted in adults with mild hypertension or normotensive males, overweight women, and patients with metabolic syndrome.	[61]
Blood Pressure, lipid profile, blood biochemistry	28 days	46 mg/day GCBE (containing 25 mg CGA), 93 mg/day (containing 50 mg CGA), or 185 mg/day (containing 100 mg CGA)	Randomized controlled trial (RCT) in mildly hypertensive subjects	[62]

**Table 3:** Food ORAC value tiers with examples.

ORAC values above 10,000	
English walnuts	13,541
Fresh oregano	13,970
Fresh peppermint	13,978
ORAC values above 7000–10,000	
Raw lentils	7282
Raw pinto beans	7779
Raw pistachio nuts	7983
ORAC values of 3000–5000	
Raw apples with skin on	3082
Raw broccoli	3083
Raw gooseberries	3277
Sweet, raw cherries	3365
Raw green apples with skin	3898
White raisins dried to 40% moisture	4188
Raw red delicious apples with skin on	4275
Raw raspberries	4882

Values derived from https://superfoodly.com/orac-values/ (accessed on 1 December 2025) [82].

**Table 4:** Ingredients in DailyColors^TM^ and their corresponding ORAC ranking.

Ingredient in DailyColors^TM^	ORAC Value (µmole Trolox Equivalents/100 g) *	ORAC *	ORAC Ranking Among Highest ORAC Foods (Out of 498) *
*Vitis vinifera* extract (fruit and leaves)	108,130	108,130	22
Ginger extract (rhizomes)	14,840	14,840	78
European elder (fruit)	14,697	14,697	79
Rosemary extract (leaves)	11,070	11,070	87
Blueberry extract (fruit)	9621	9621	94
Black currant (fruit)	7957	7957	108
Pomegranate extract (fruit)	4479	4479	145
*Malus pumila* extract (fruit)	3049	3049	179
*Coffea arabica* extract (seed)	2780	2780	182
*Beta vulgaris* (root)	1776	1776	245
*Brassica oleracea* var. *acephala* (leaf)	1770	1770	247
*Citrus paradisi* extract (fruit)	1548	1548	263
*Olea europaea* extract(fruit)	1010	1010	310
*Allium cepa* extract (bulb)	913	913	323
Carrot (root)	697	697	360
*Lycopersicon esculentum* (fruit)	3870	387	412

* Values were derived from https://superfoodly.com/orac-values/(accessed on 1 December 2025) [82].

**Table 5:** DailyColors^TM^ supplementation resulted in dose-dependent improvements in memory age from baseline (T0) to end of study (TF).

Group	Average of Memory Age T0	Average of Memory Age TF
High dose (n = 49)	64.98	60.79
Low dose (n = 39)	64.61	60.84
Placebo (n = 39)	64.21	60.57
Total average	64.63	60.73

## Data Availability

The original contributions presented in this study are included in the article. Further inquiries can be directed to the corresponding author.
